# DEVELOPING A PROTOCOL TO ASSESS EXPOSURE TO ENVIRONMENTAL POLLUTANTS AMONG OLDER ADULT WOMEN IN SOUTHEAST MICHIGAN

**DOI:** 10.1093/geroni/igad104.3269

**Published:** 2023-12-21

**Authors:** Jillian Baker, Sung Kyun Park, Kerry Richards-McCullough, Andrew Hoover, Carrie Karvonen-Gutierrez

**Affiliations:** University of Michigan School of Public Health, Ann Arbor, Michigan, United States; University of Michigan School of Public Health, Ann Arbor, Michigan, United States; University of Michigan School of Public Health, Ann Arbor, Michigan, United States; University of Michigan School of Public Health, Ann Arbor, Michigan, United States; University of Michigan School of Public Health, Ann Arbor, Michigan, United States

## Abstract

Environmental pollutants, including PFAS, phthalates, and parabens, contribute to the development of age-related diseases like cancer, cardiometabolic disease, and neurodegenerative diseases. Unfortunately, these chemicals are common in everyday exposures, including drinking water, personal care products (PCPs), non-stick cookware, and food packaging. Exposures to these pollutants may be high in communities that have encountered structural disadvantages; neighborhood disinvestment and decaying infrastructure can make exposures to these pollutants more likely, thus increasing the burden of age-related disease in these communities. Interventions that reduce exposures to these chemicals may prevent or delay the onset of age-related disease, especially in groups experiencing poorer health outcomes because of structural racism and discrimination. To explore the feasibility of a behavior change intervention for older adults that reduces exposure to harmful chemicals in PCPs, a protocol was developed to: 1) Assess attitudes, knowledge, and behaviors related to environmental pollutants via questionnaire; 2) Characterize individual-level ‘exposomes’, the unique mixture of chemicals a person encounters in their everyday lives, by wearing a silicone wristband; 3) Measure environmental exposures in serum. Data collection will begin in September 2023 among older adult women who have participated in the Study of Women’s Health Across the Nation (SWAN) at the Michigan site. Older adults have accrued environmental exposures across their lifetimes, likely in a way that reflects patterns of structural disadvantage. While structural solutions are necessary to address issues of health equity, behavior change efforts can work in tandem to reduce harmful environmental exposures to improve the health of older adults.

